# Supplementary material for: Unraveling chirality transfer mechanism by structural isomer-derived hydrogen bonding interaction in 2D chiral perovskite
Source: Nat Commun. 2023 May 30;14:3124. doi: 10.1038/s41467-023-38927-2 (PMC10229653; doi:10.1038/s41467-023-38927-2)
Supplement: Supplementary file 1 — Supplementary Information [file 41467_2023_38927_MOESM1_ESM.docx]

Supplementary Information for

Unraveling chirality transfer mechanism by structural isomer derived hydrogen bonding interaction in 2D chiral perovskite

Jaehyun Son^1,2^, Sunihl Ma^1,2,3^, Young-Kwang Jung^1^, Jeiwan Tan^1^, Gyumin Jang^1^, Hyungsoo Lee^1^, Chan Uk Lee^1^, Junwoo Lee^1^, Subin Moon^1^, Wooyong Jeong^1^, Aron Walsh^4^ and Jooho Moon^1^*

^1^Department of Materials Science and Engineering

Yonsei University

50 Yonsei-ro Seodaemun-gu, Seoul, 03722, Republic of Korea

^2^These authors contributed equally: Jaehyun Son, Sunihl Ma

^3^Current address : Department of Chemical Engineering, University of Michigan, Ann Arbor

MI 48109, United States

^4^Department of Materials

Imperial College London

London SW7 2AZ, UK

* Corresponding author: jmoon@yonsei.ac.kr

This PDF file includes:

Supplementary Figure 1 to 26

Supplementary Note 1

Supplementary Table 1 to 4

References


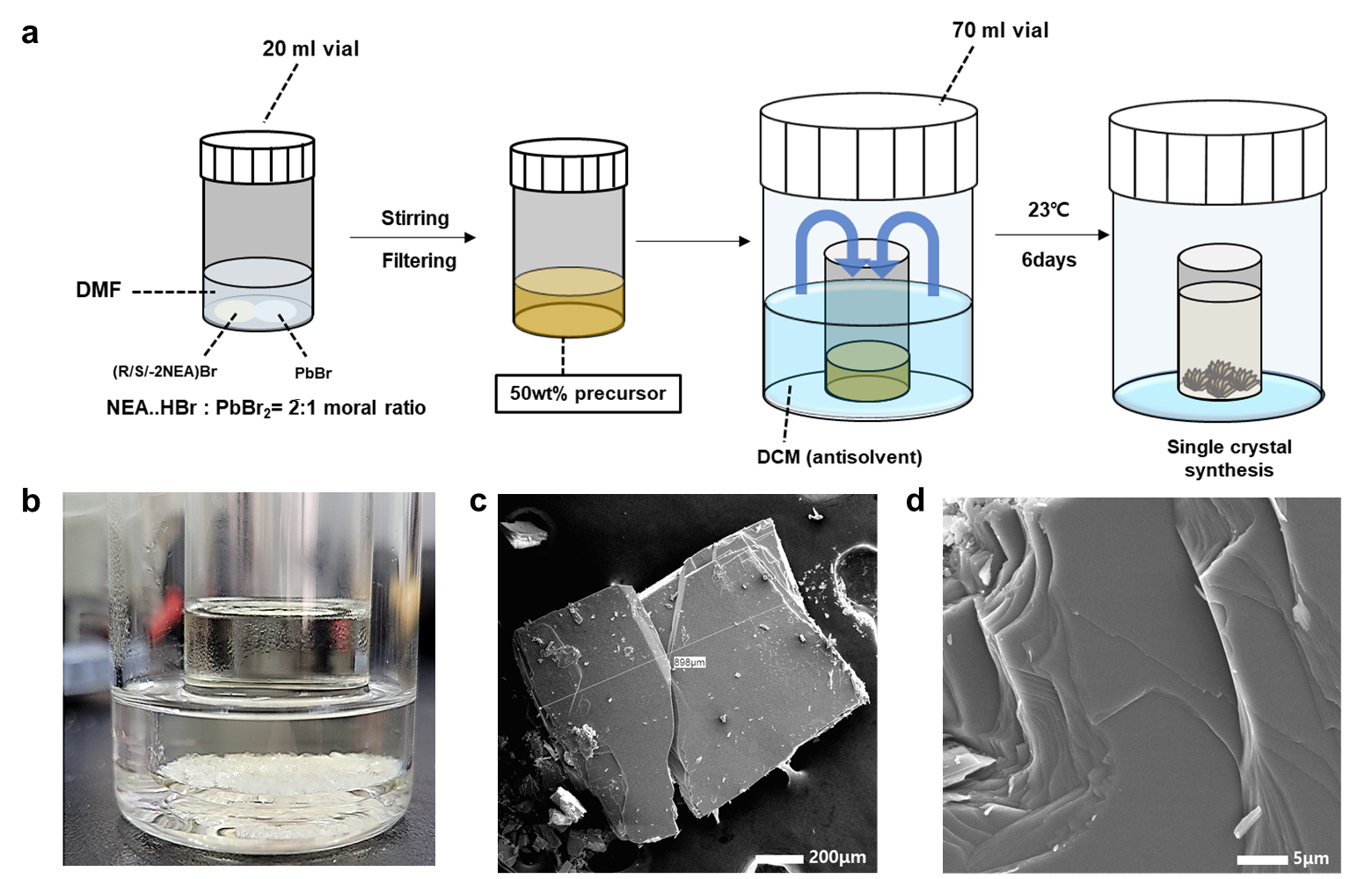


**Supplementary Figure 1. a,** Schematic illustration of AVC single crystal synthesis method. **b,** Photograph of synthesized single crystal after 6 days **c and** **d,** SEM images of synthesized single crystals.


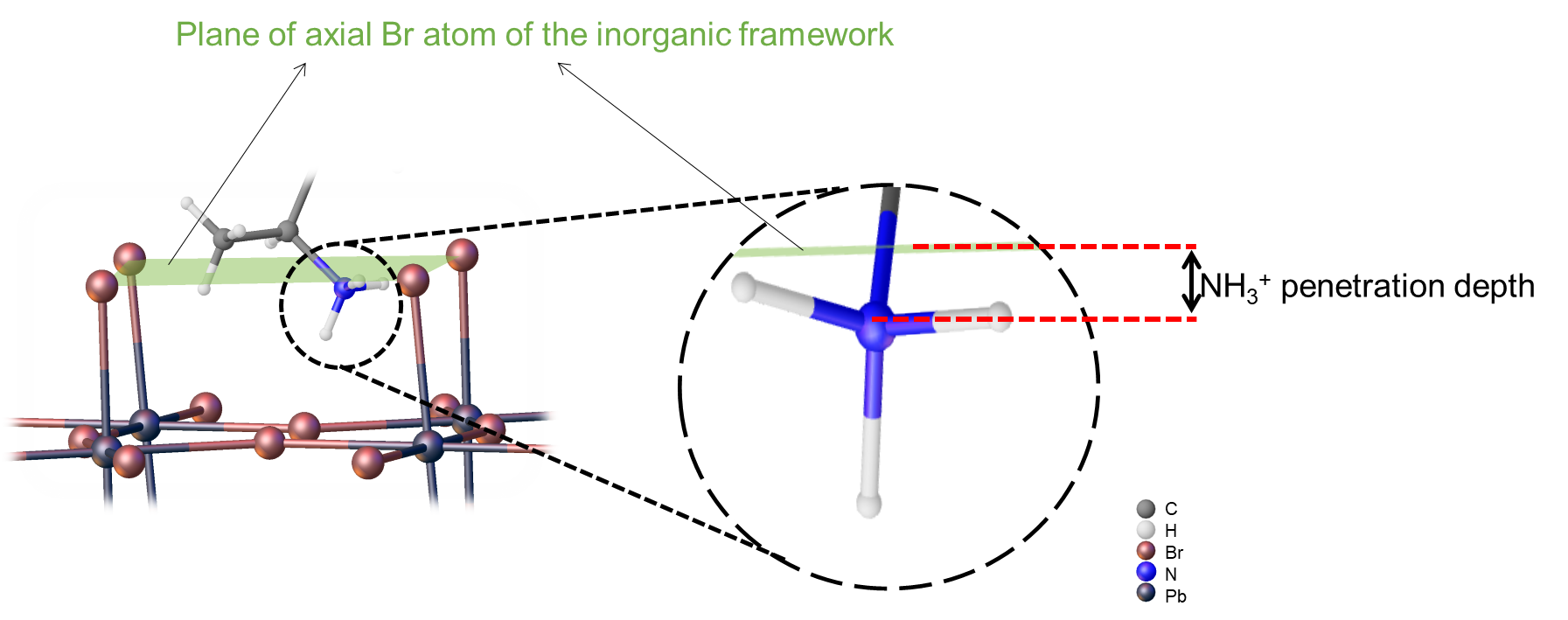


**Supplementary Figure 2.** Measurement method for NH_3_^+^ penetration depth. The distance was calculated using OLEX 2 software.


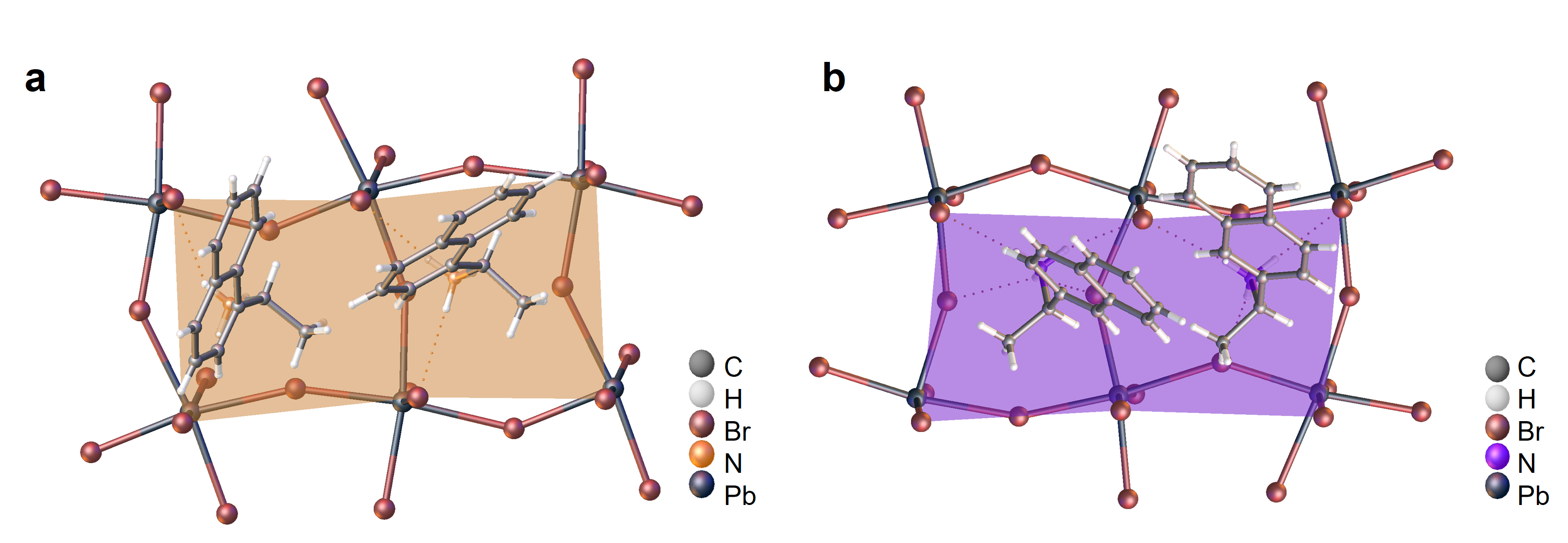


**Supplementary Figure 3. Crystal structures of NEA isomer OIHPs viewed from [001] direction.**


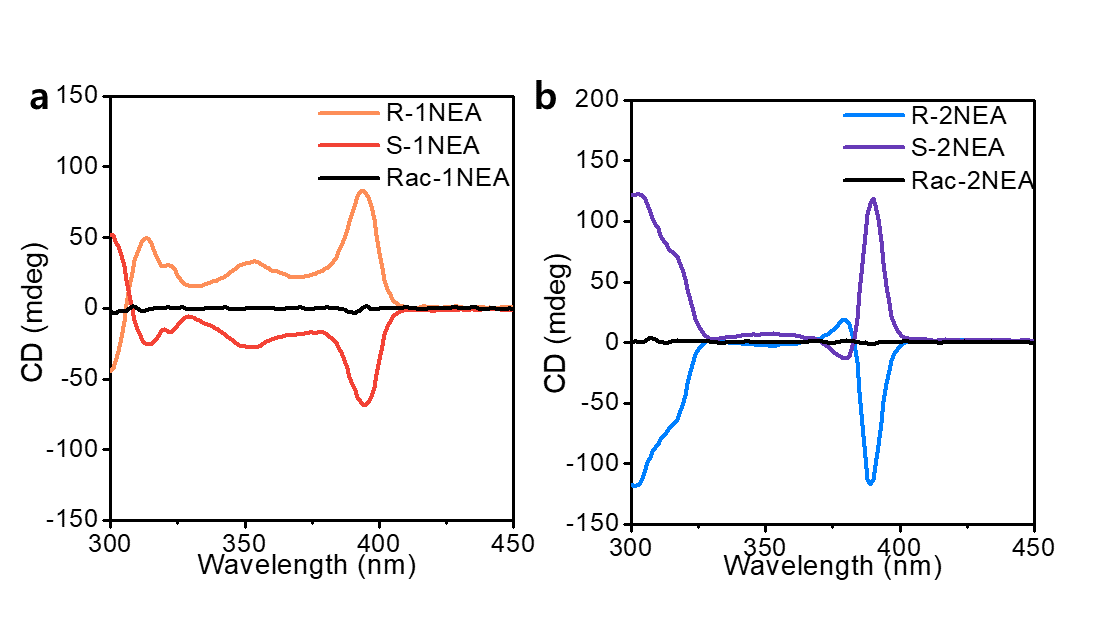


**Supplementary Figure 4. CD spectra of the R-/S-/Rac-NEA OIHPs. a,** CD spectra of the (R/S/rac-1NEA)­_2_­PbBr­_4_ thin-films and **b,** CD spectra of the (R/S/Rac-2NEA)_2_PbBr_4_ ­thin-films. Source data are provided as a Source Data file.


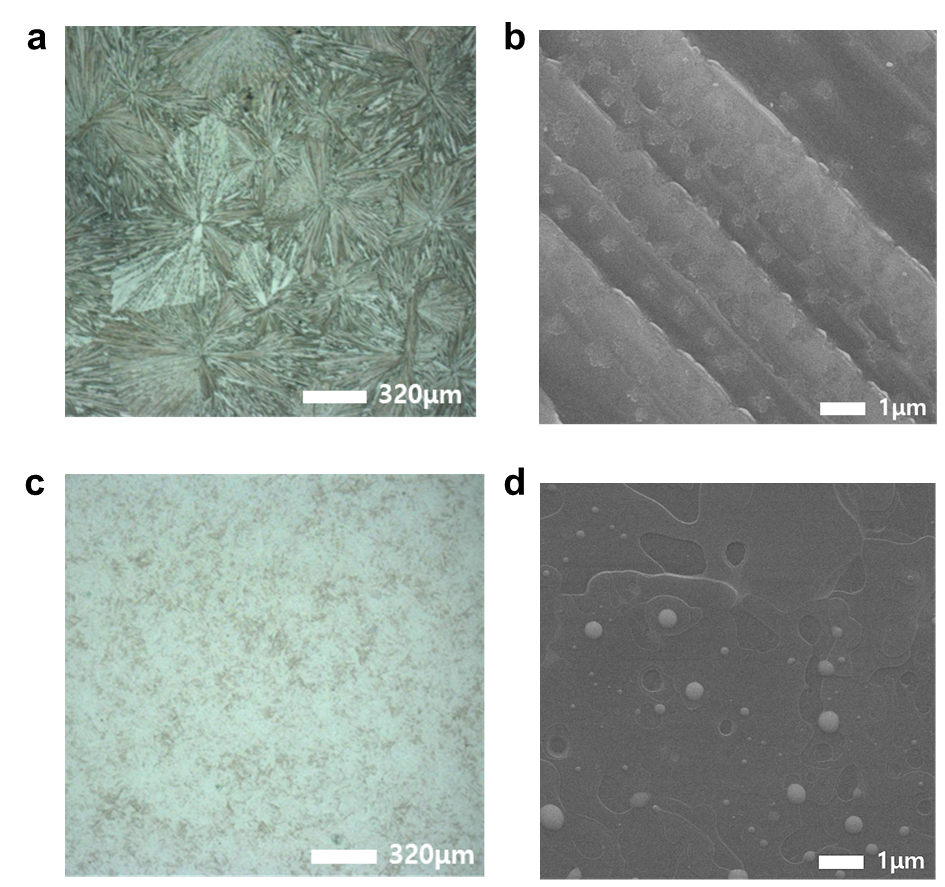


**Supplementary Figure 5. Surface morphology characteristics of the NEA OIHPs thin-films synthesized without MABr additive. a,** Optical microscope (OM) image of *R*-1NEA thin film and **b**, SEM image of *R*-1NEA thin film. **c,** OM image of *R*-2NEA thin-film and **d,** SEM image of *R*-2NEA thin-film.

**
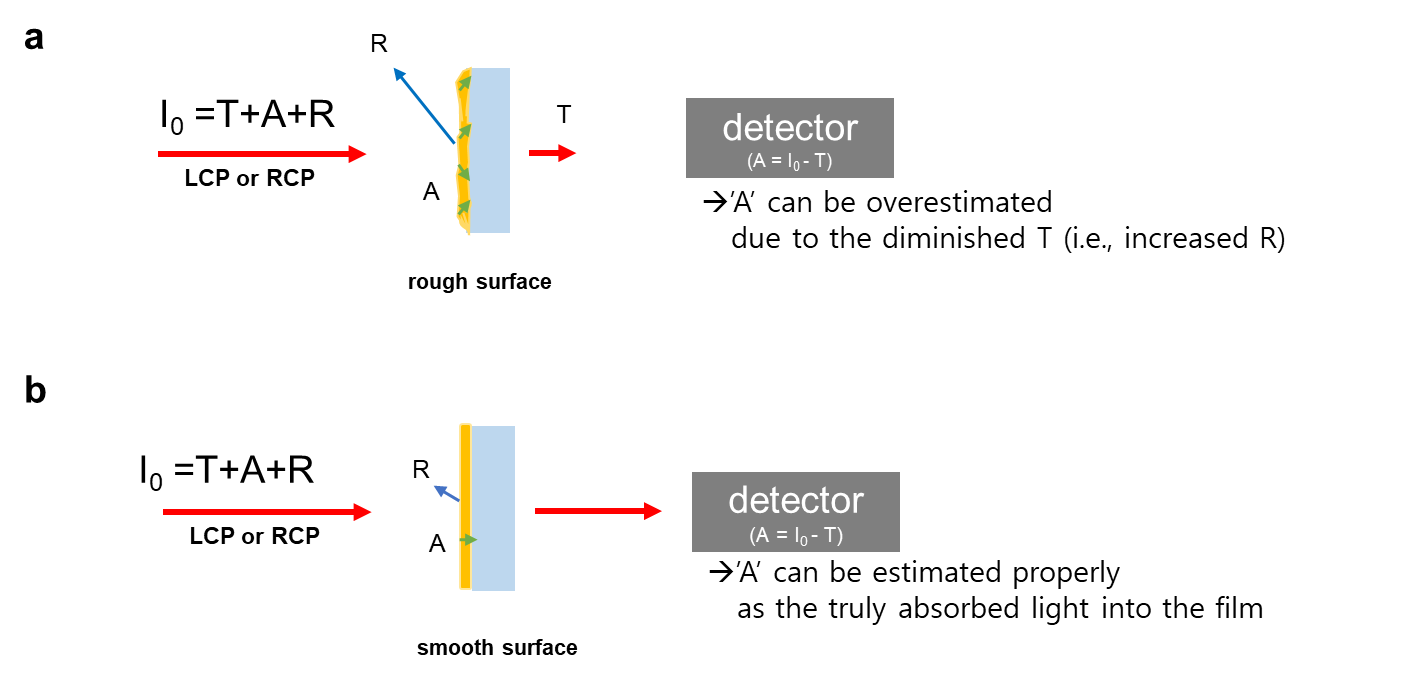
**

**Supplementary Figure 6. Schematic illustration of transmission CD spectra analysis.** The symbol R means reflected light, A indicates absorbed light due to the refraction on the film surface, T represents transmitted light, and I­_0_ stands for the initial irradiated CPL light from the light source. **a,** Case of CPL irradiation on the rough thin-film surface, and **b,** case of smooth thin-film. In the case of rough surface, the probability that the irradiated CPL from the light source is refracted into thin-film or reflected to surroundings (significant R). However, the detectors recognize the absorption a by subtracting transmitted light (the light that reached the detector) from I_0 ­_­(*i.e.,* A = I_0_ - T), without considering the R. Thus, the degree of CD (*i.e*, degree of CPL absorption into the thin-film) can be overestimated when the light is transmitted through the rough surface with significant R owing to the diminished transmitted light^1^.


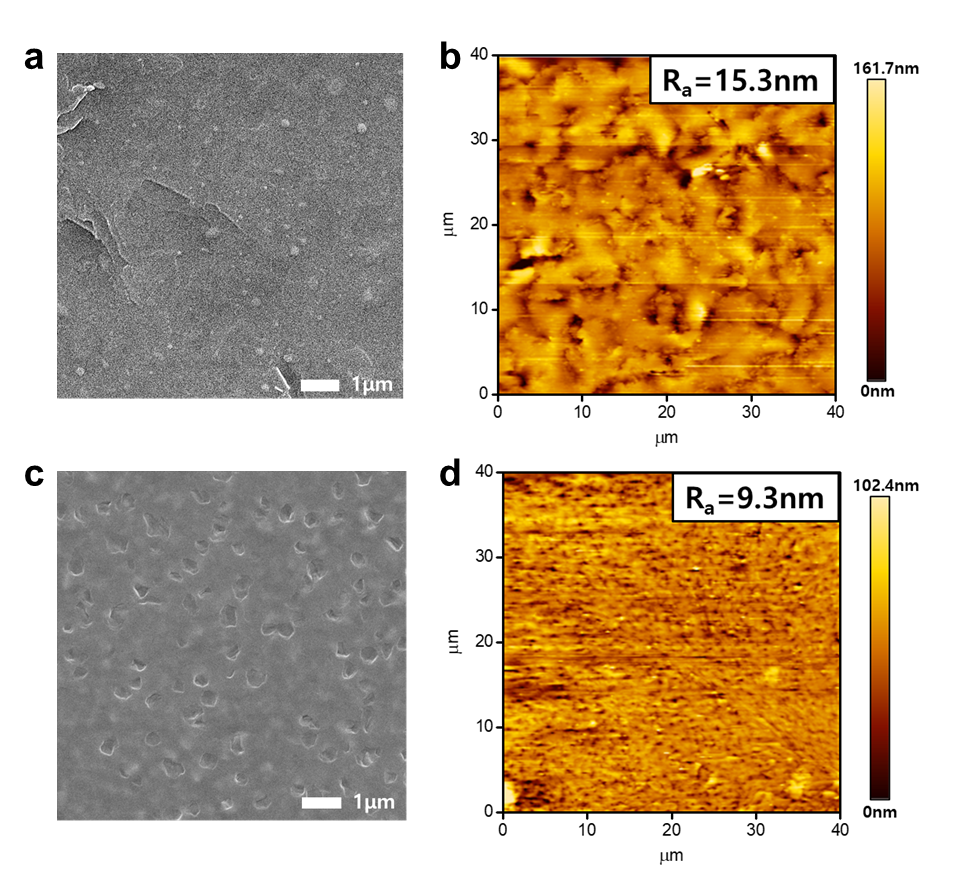


**Supplementary Figure 7. Surface morphology characteristics of the NEA OIHPs thin-films synthesized with 10 mol% of MABr additive. a,** SEM image and **b,** AFM topography images of controlled *R*-1NEA. **c,** SEM image and **d,** AFM topography images of controlled *R*-2NEA. The controlled films showed a similar roughness average (R_a_) of 15.3 nm and 9.3 nm for *R*-1NEA and *R*-2NEA, respectively.


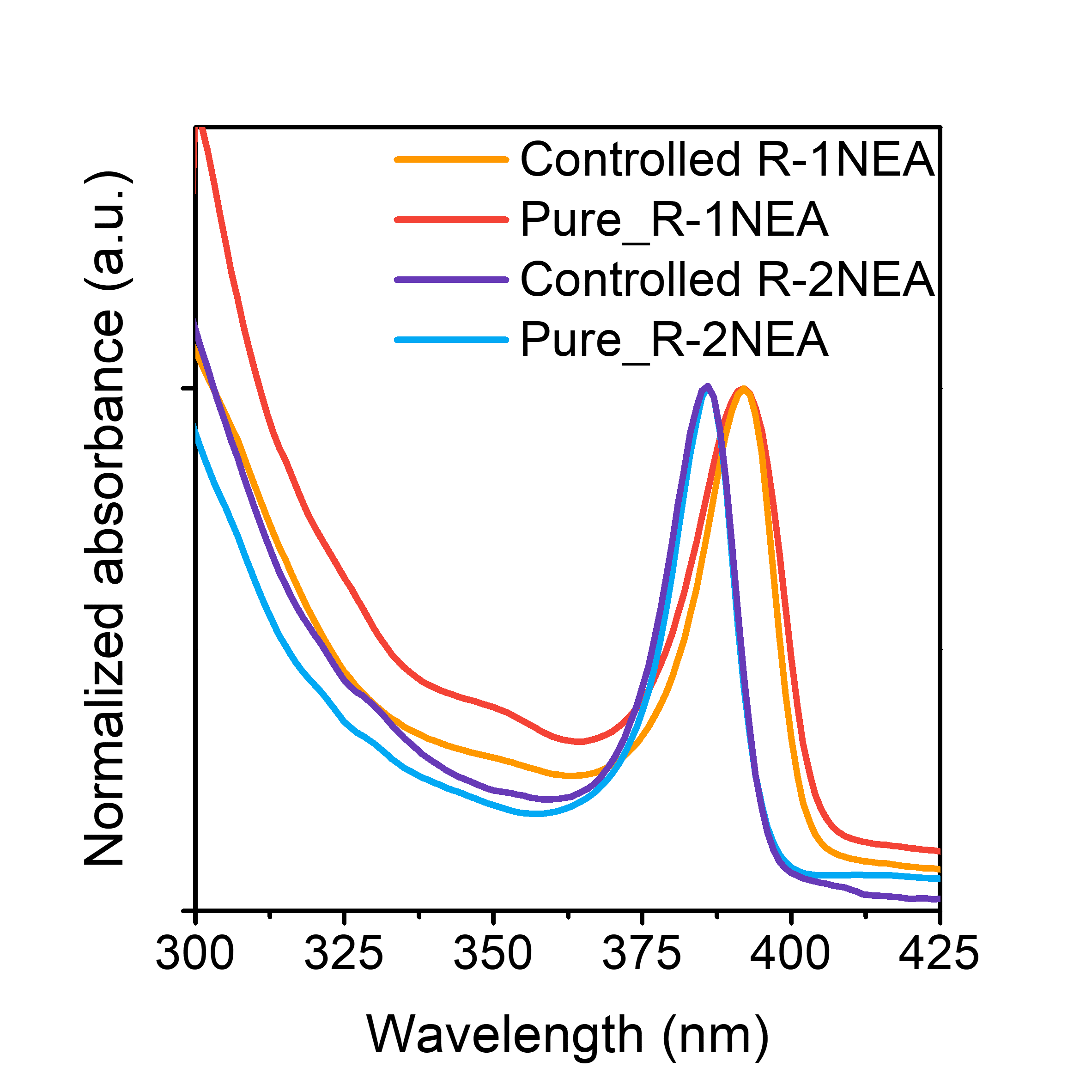


**Supplementary Figure 8.** Normalized absorbance spectra for controlled NEA OIHPs and pure NEA OIHPs thin-film. Source data are provided as a Source Data file.

**Supplementary Note 1**

**Details on the fitting procedure**

We adopted the suggested CD spectra deconvolution protocol from the previous literature.^2,3^ The absorbance spectra of chiral OIHPs thin film was fitted by a sum of Gaussian using the least square means with fitting parameters of (𝐴_0i_, 𝜆_0i_ , 𝜎­_i_). The initial values were estimated from the absorption center, and the width was approximated from the second derivative maxima with coefficients of determination : R^2^ > 0.96. The CD spectrum fitting was mainly conducted near the first excitonic band edge for the controlled R-NEA OIHPs spectra.


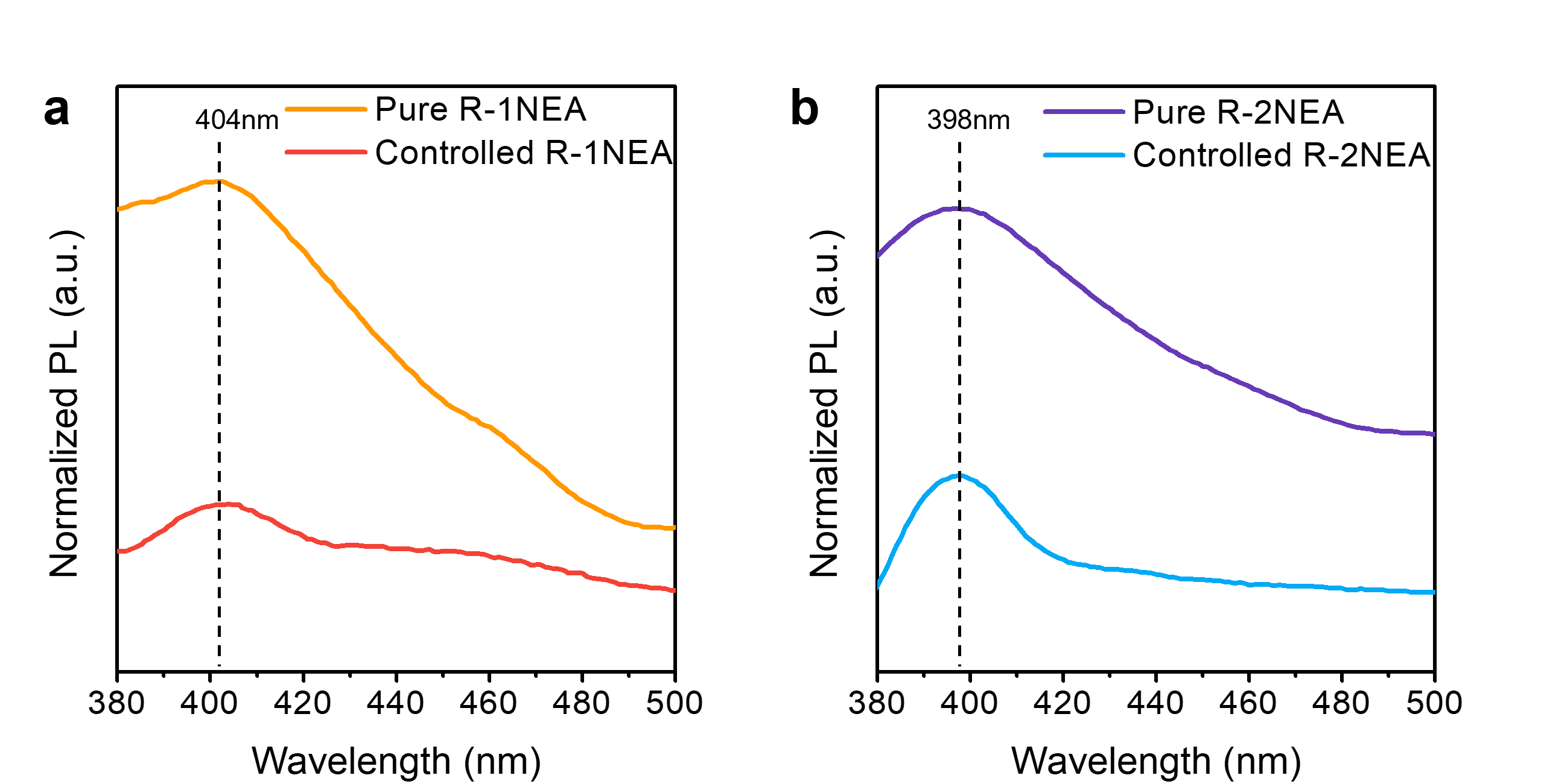


**Supplementary Figure9. Steady-state PL spectra for NEA isomer OIHP thin films.** The excitonic PL peaks are observed at **a**, 404 nm for R-1NEA and **b**, 398 nm for R-2NEA, respectively. Source data are provided as a Source Data file.

**
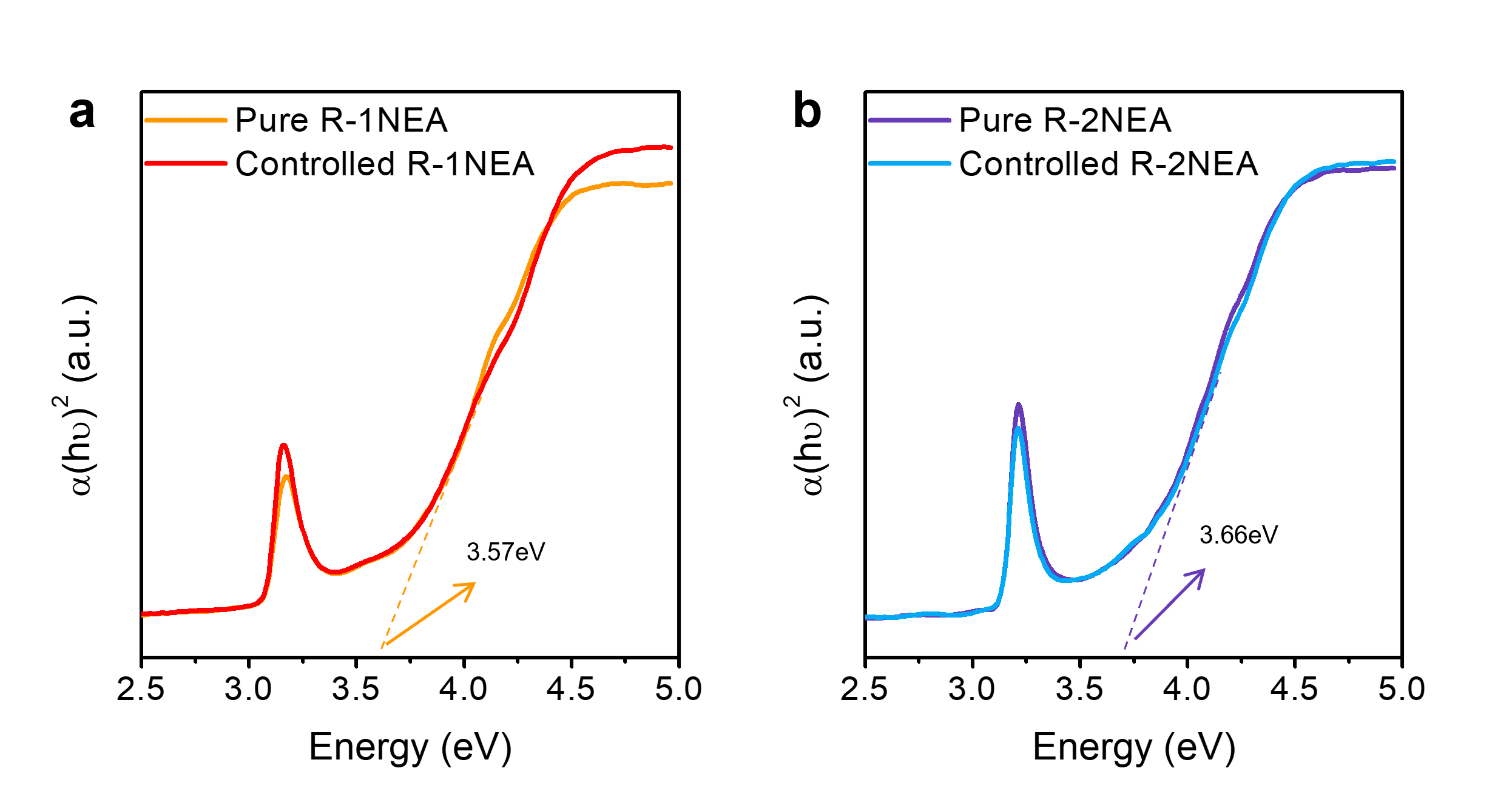
**

**Supplementary Figure 10. Tauc plot of the NEA and controlled NEA isomer OIHP thin films. a,** Tauc plot for R-1NEA and **b,** R-2NEA OIHPs. Source data are provided as a Source Data file.

**
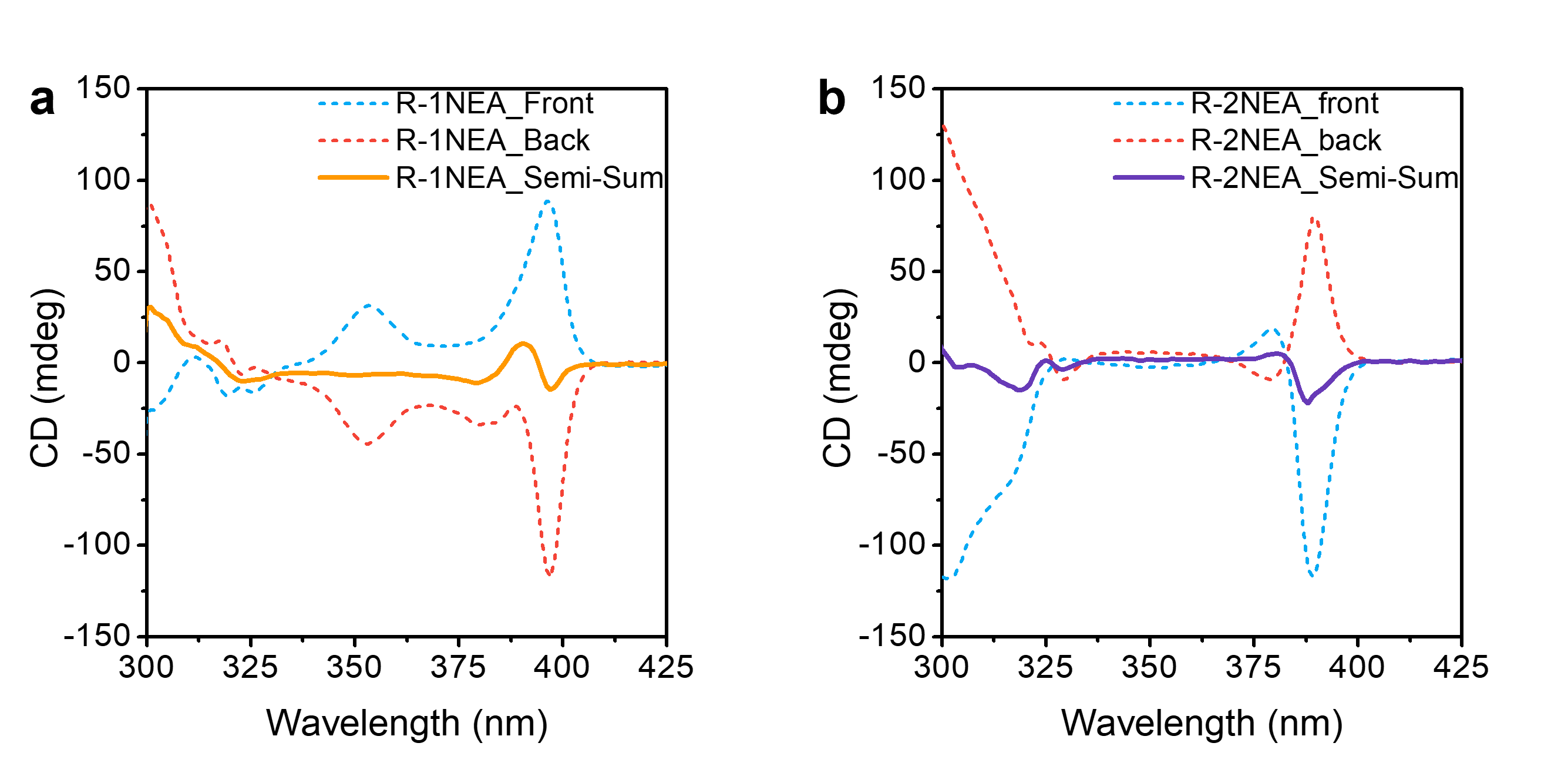
**

**Supplementary Figure 11. The CD spectra measured under sample flipping condition.** The dashed sky-blue and red lines represent the measured CD spectra from front and back side of the films, respectively. The solid orange and purple lines indicate the CD_true_, which is calculated by taking semi-sum. Source data are provided as a Source Data file.

**
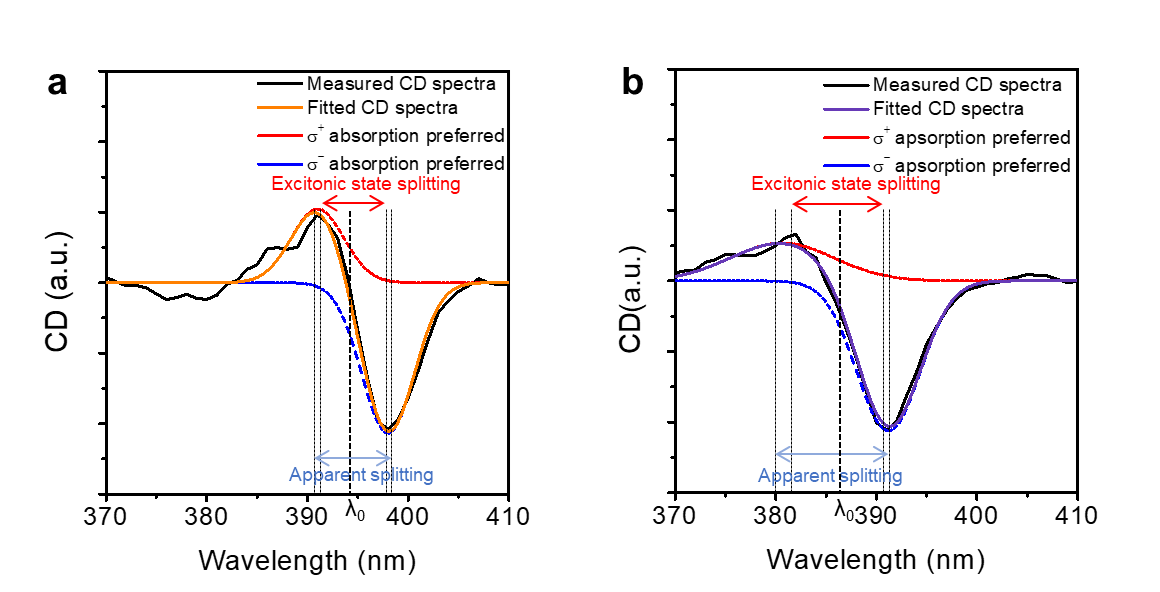
**

**Supplementary Figure 12. Gaussian fitting of the CD spectra. a,** Deconvolution result for **a,** controlled R-1NEA and **b,** controlled R-2NEA OIHPs. The black solid black line result from the experimentally measured CD spectra from the chiral NEA structural isomer OIHPs. The red and blue dot-line, which indicate the absorption of RCP and LCP, respectively, are obtained by Gaussian fitting. Source data are provided as a Source Data file.

**
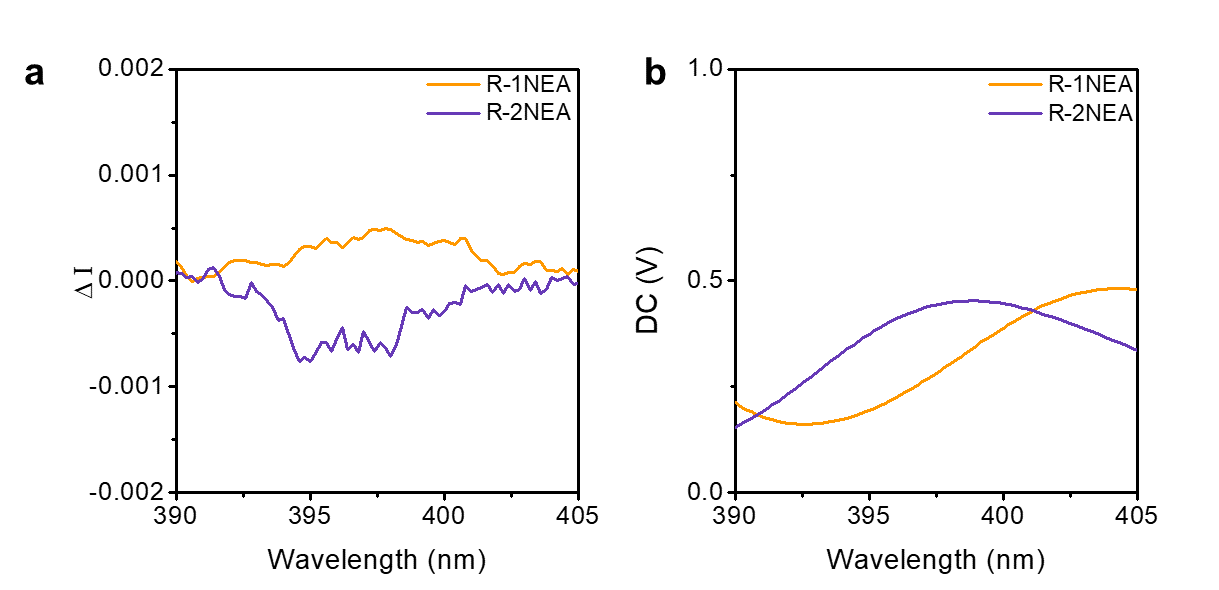
**

**Supplementary Figure 13. CPL measurement for R-NEA structural isomer OIHPs. a,** ΔI represent the intensity difference between RCP and LCP. **b**, The maximum DC voltage was set to about 0.5V. The wavelength of maximum DC voltage is 404nm for R-1NEA OIHPs and 399nm for R-2NEA OIHPs. Source data are provided as a Source Data file.

**
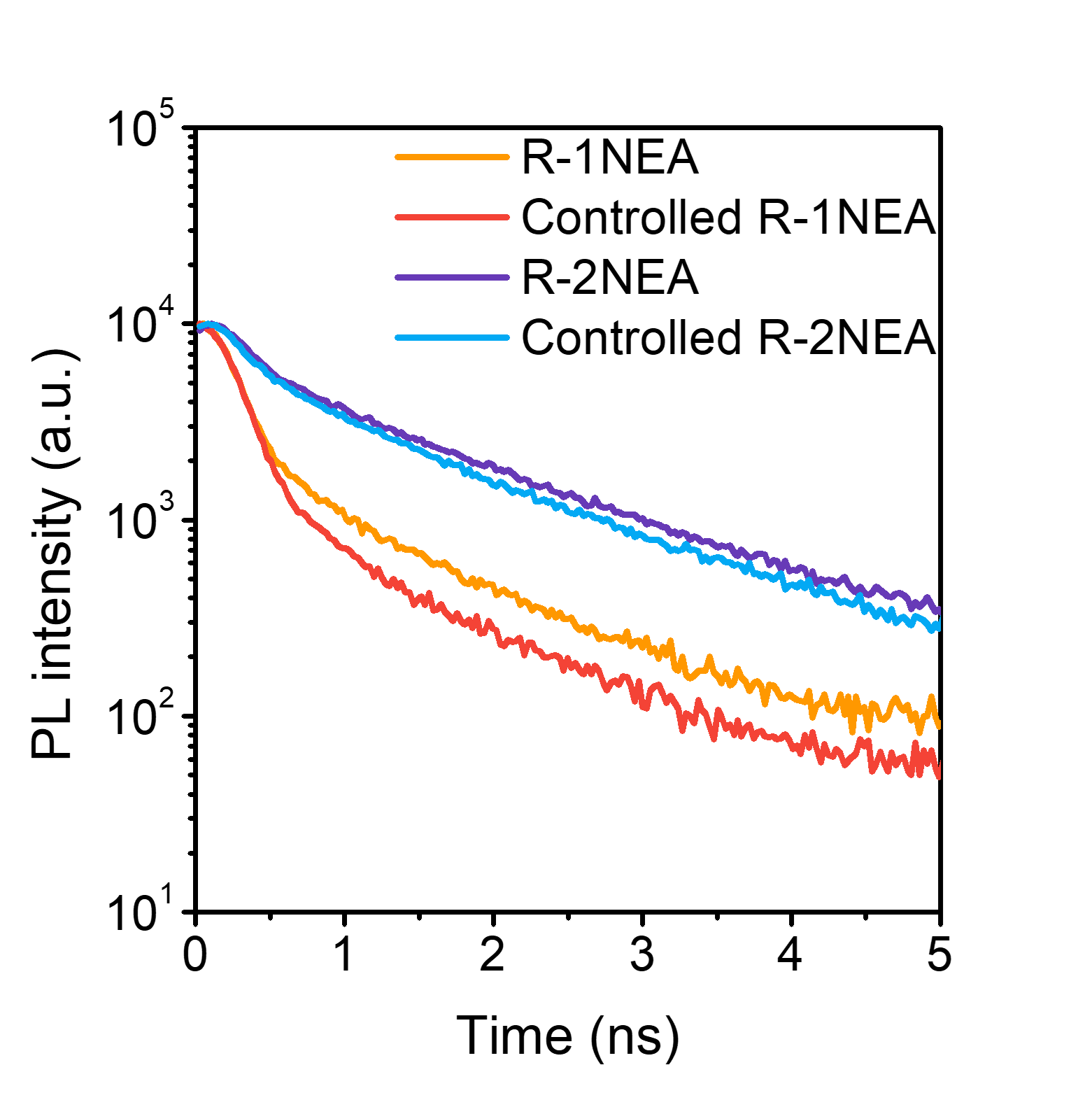
**

**Supplementary Figure 14. TRPL spectroscopy of R-NEA and controlled R-NEA OIHP thin films measured with excitation wavelength of 371nm.** The traces fitted to a biexponential decay function of y (t) = A_1_ exp (-t/τ_1_) + A_2_ exp (-t/τ_2_) with adj. R^2^ > 0.99. Source data are provided as a Source Data file.

**
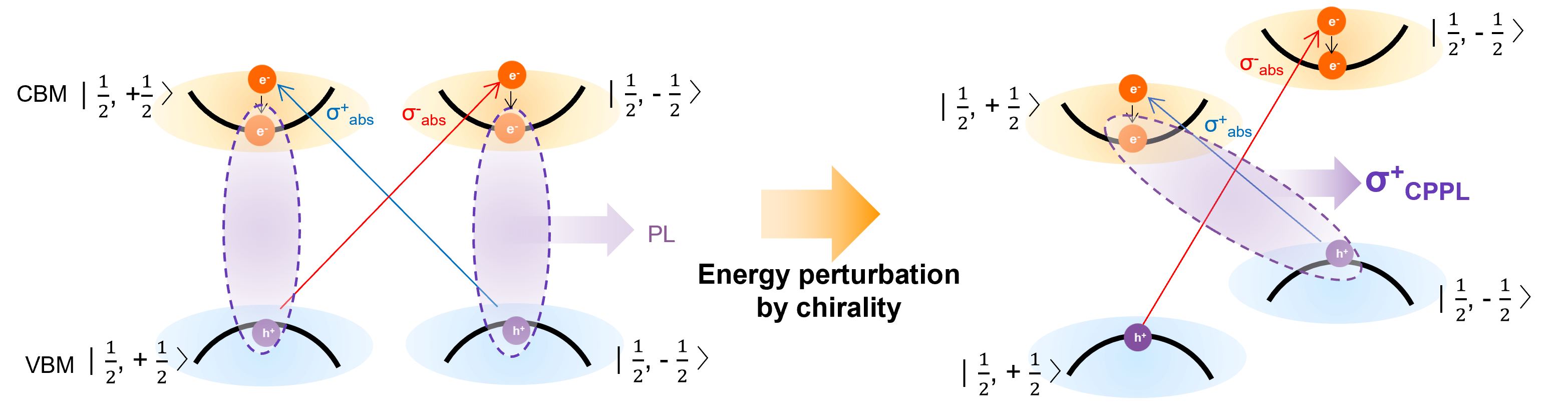
**

**Supplementary Figure 15. Schematic illustration of energy diagram perturbation by chirality and the CPPL emission mechanism.** The conduction band minimum (CBM) and valence band maximum (VBM) are composed of the states with total angular moment quantum number (J = $\frac{\text{1}}{\text{2}}$) and magnetic quantum number (m_s_) of$\text{ ±}\frac{\text{1}}{\text{2}}$ according to the spin-state of electrons. The σ^+^_abs_ and σ^-^_abs_ indicate the absorption of the RCP (+) and LCP(-), respectively. The σ^+^_abs_ corresponds to the excitonic transition where the magnetic quantum number changes from $\text{-}\frac{\text{1}}{\text{2}}$to$\text{+}\frac{\text{1}}{\text{2}}$(vice versa for σ^-^_abs_). The excitonic states can be perturbated when the chirality is transferred to the OIHP lattice. In this case, the energy states with up-spin state (m_s_=$\text{+}\frac{\text{1}}{\text{2}}$) and down-spin state (m_s_ =$\text{-}\frac{\text{1}}{\text{2}}$) are no longer identical. Therefore, the photon emitted from the chiral OIHPs can be spin-polarized.


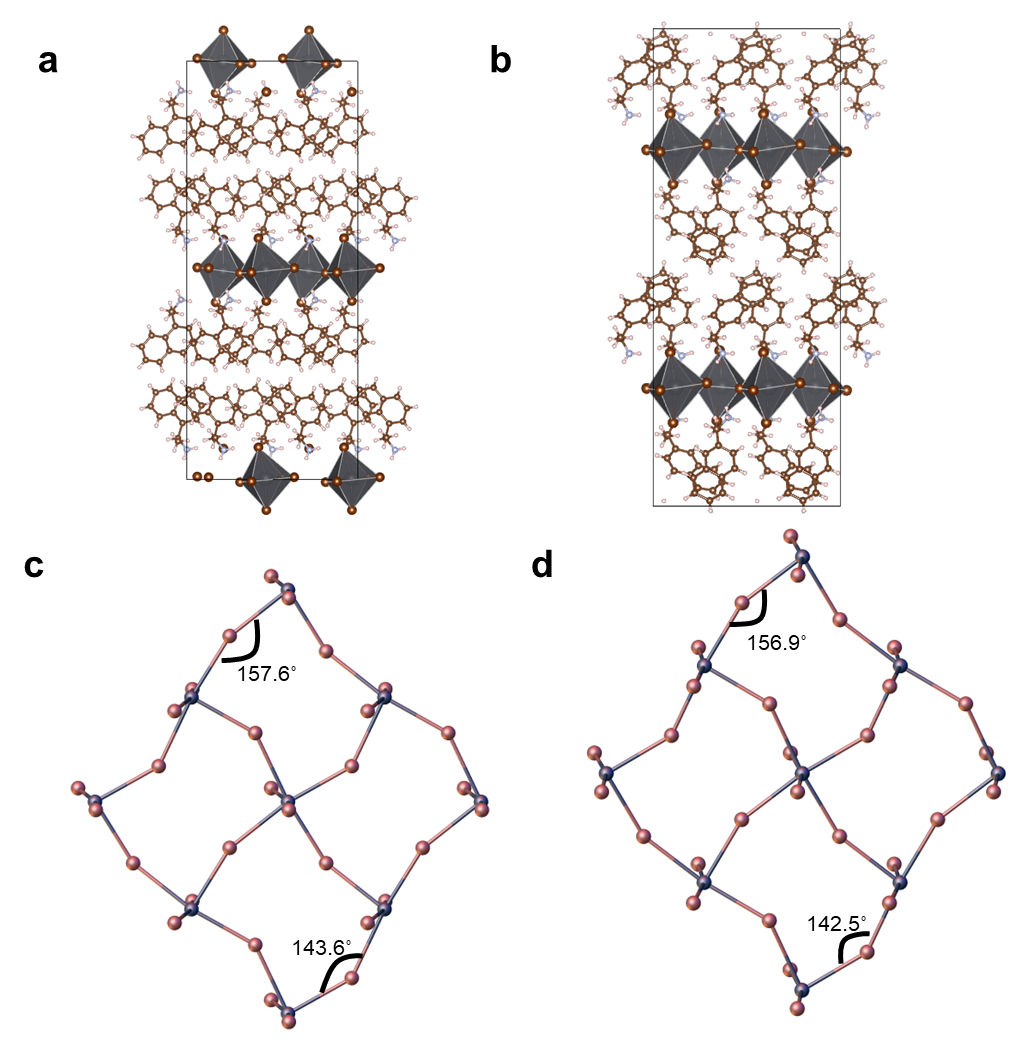


**Supplementary Figure 16. Crystal structure of the S-NEA structural isomer OIHPs viewed from various directions. a** and **b,** Crystal structure of S-NEA structural isomer OIHPs viewed from [100] direction; **a**, (S-1NEA)_2_PbBr_4_ **b,** (S-2NEA)_2_PbBr_4_. **c** and **d**, Inorganic layer structure viewed from [001] direction; **c, (**S-1NEA)_2_PbBr_4_ and **d,** (S-2NEA)_2_PbBr_4_. Brown and dark spheres denote Br and Pb atoms, respectively. C, H, and N atoms are omitted for clarity. **e**, Normalized absorbance spectra for (S-NEA)_2_PbBr_4_.


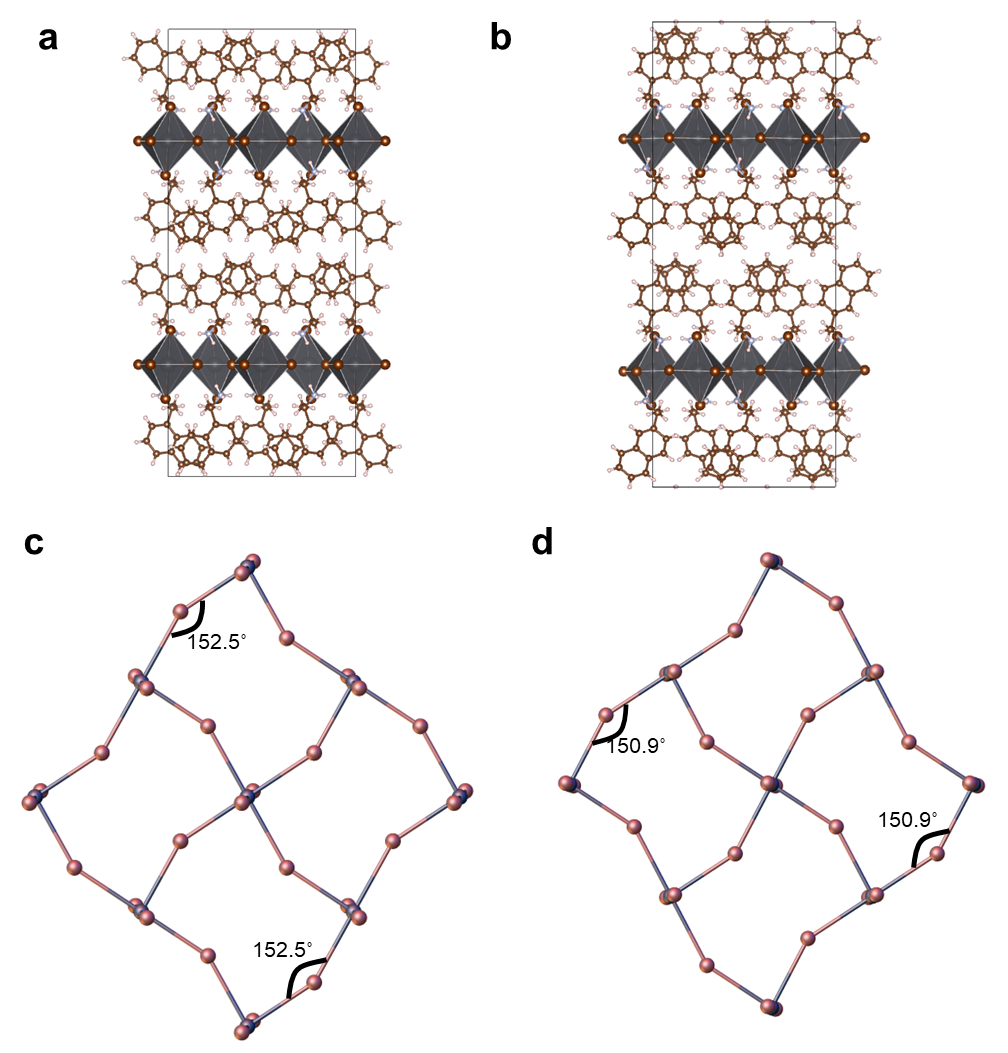


**Supplementary Figure 17. Crystal structure of the racemic-NEA structural isomer OIHPs viewed from various directions. a** and **b,** Crystal structure of racemic-NEA structural isomer OIHPs viewed from [100] direction; **a,** (Rac-1NEA)_2_PbBr_4_ and **b,** (Rac-2NEA)_2_PbBr_4_. **c** and **d**,Inorganic layer structure viewed from [001] direction; **c, (**Rac-1NEA)_2_PbBr_4_ and **d,**(Rac-2NEA)_2_PbBr_4_. Brown and dark spheres denote Br and Pb atoms, respectively. C, H, and N atoms are omitted for clarity.


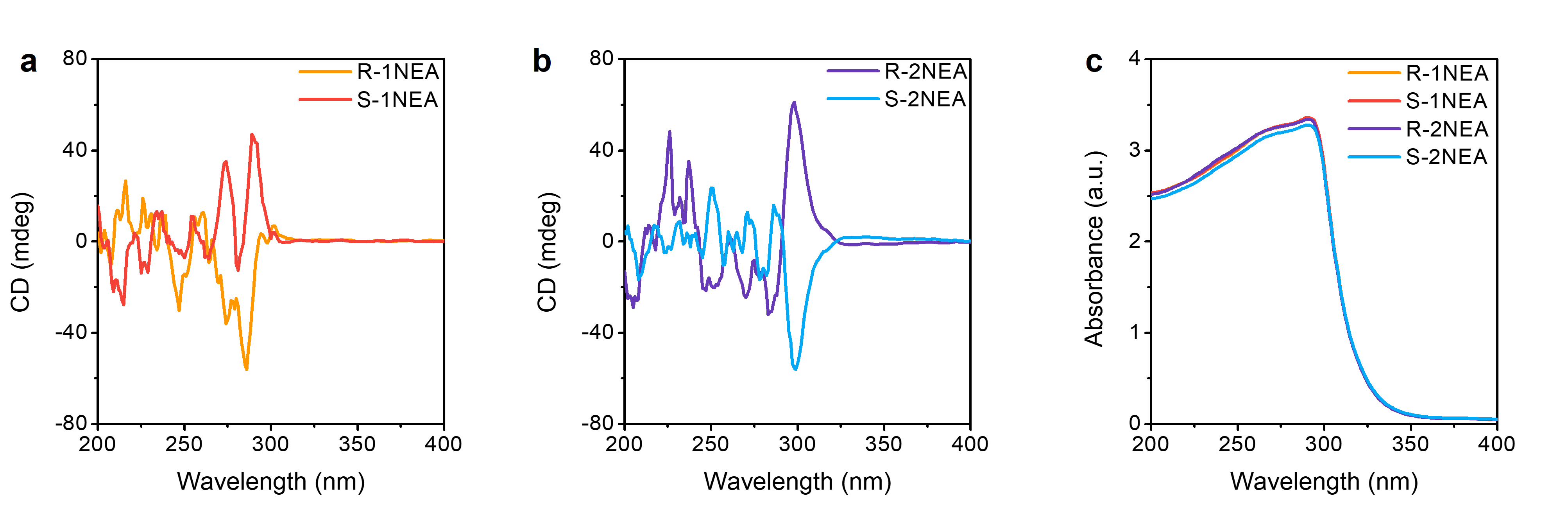


**Supplementary Figure 18. CD and absorbance spectra of chiral NEA molecules. a,** CD spectra for R/S-1NEA molecules and **b,** R/S-2NEA molecules. **c,** Absorbance spectra for NEA isomer molecules. Source data are provided as a Source Data file.


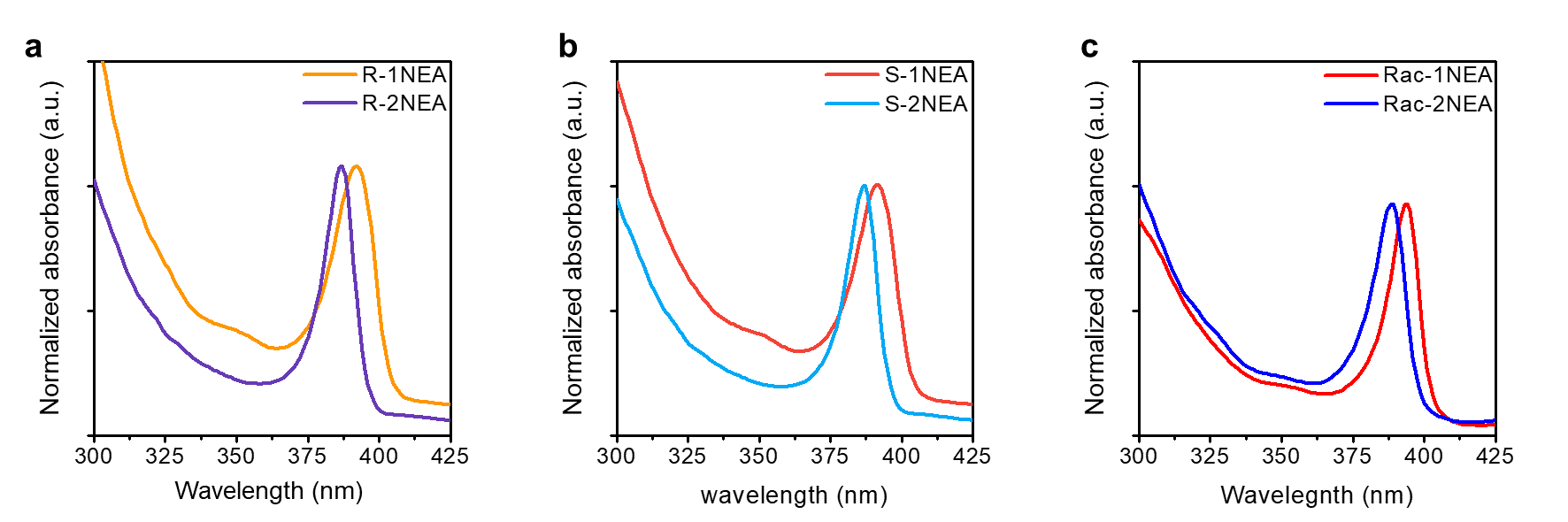


**Supplementary Figure 19.** Normalized absorbance spectra for **a,** R-NEA **b,** S-NEA, **c,** Rac-NEA structural isomer OIHPs. Source data are provided as a Source Data file.

**
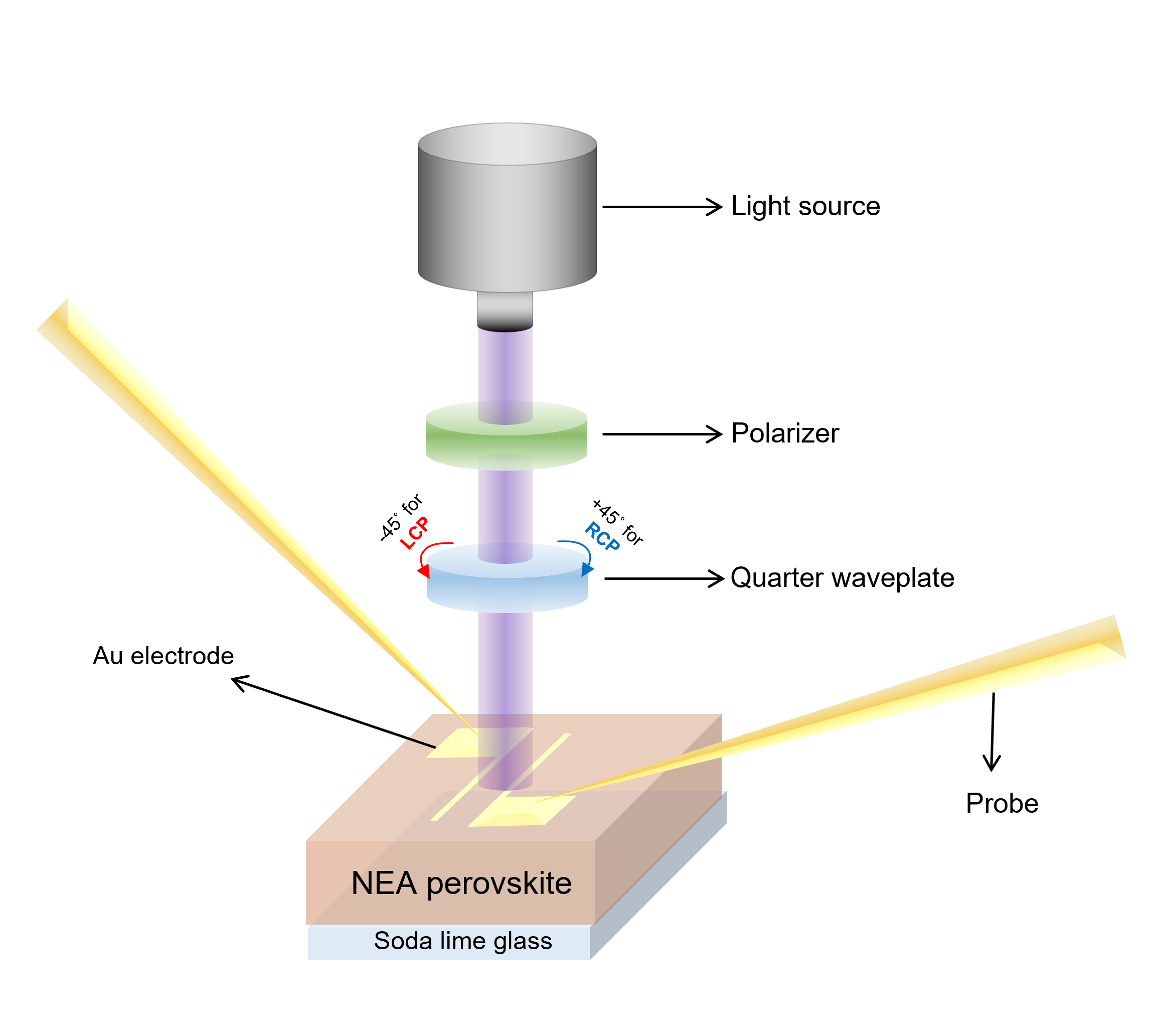
**

**Supplementary Figure 20.** **Schematic illustration of proof-of-concept planar type CPL-PDs application.** The channel between neighboring electrodes had a length of 70 μm. The light was generated by LEDs and laser with various wavelengths of 365 nm, 385 nm, and 400 nm. The unpolarized light was converted to circularly polarized light by using linear polarizer and quarter-waveplate (Thorlabs, LPVISA050).


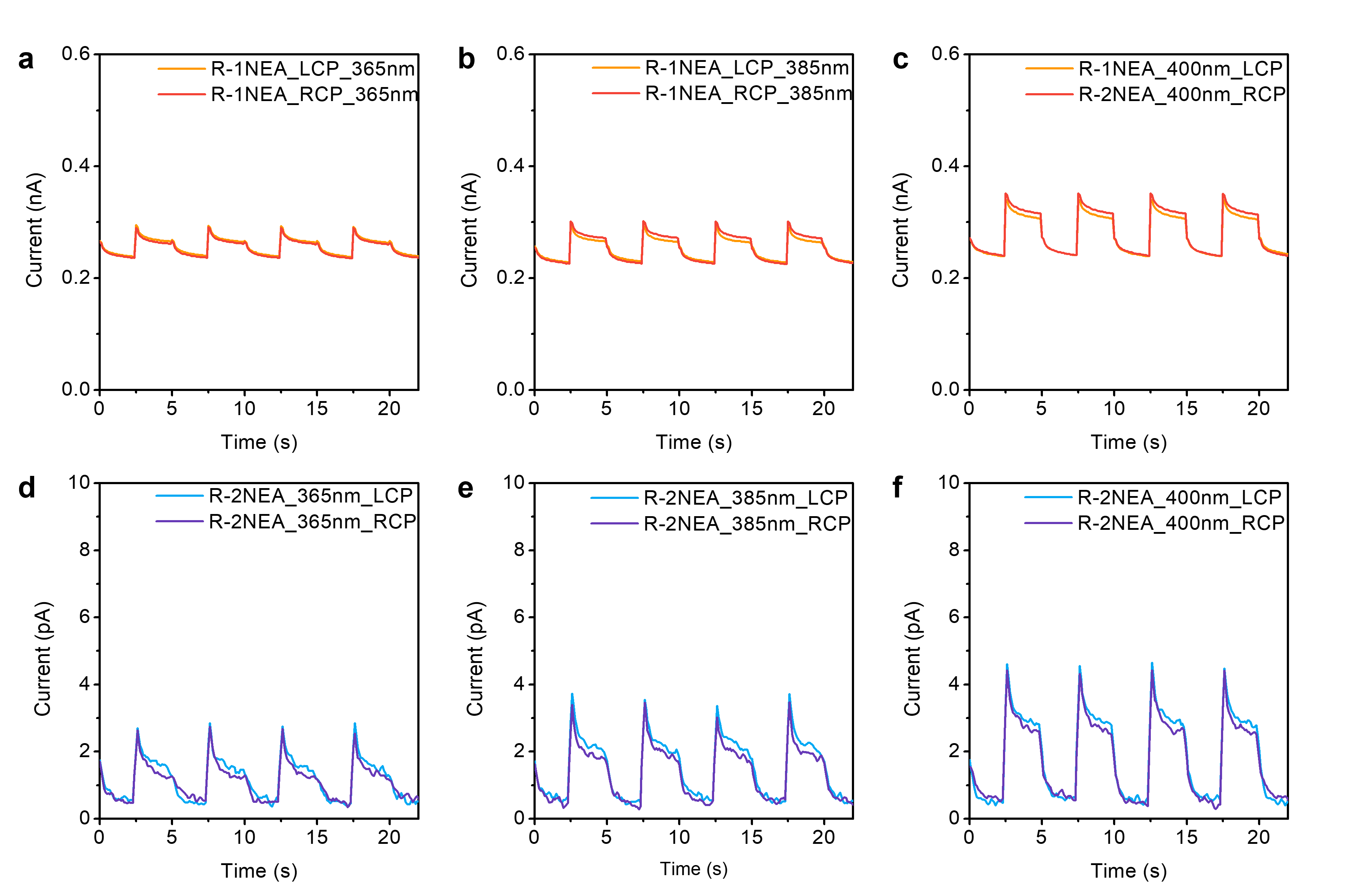


**Supplementary Figure 21. Photocurrent-time curve under LCP and RCP with the varied CPL wavelength.** The photocurrent was measured at an applied voltage of 4V. Source data are provided as a Source Data file.


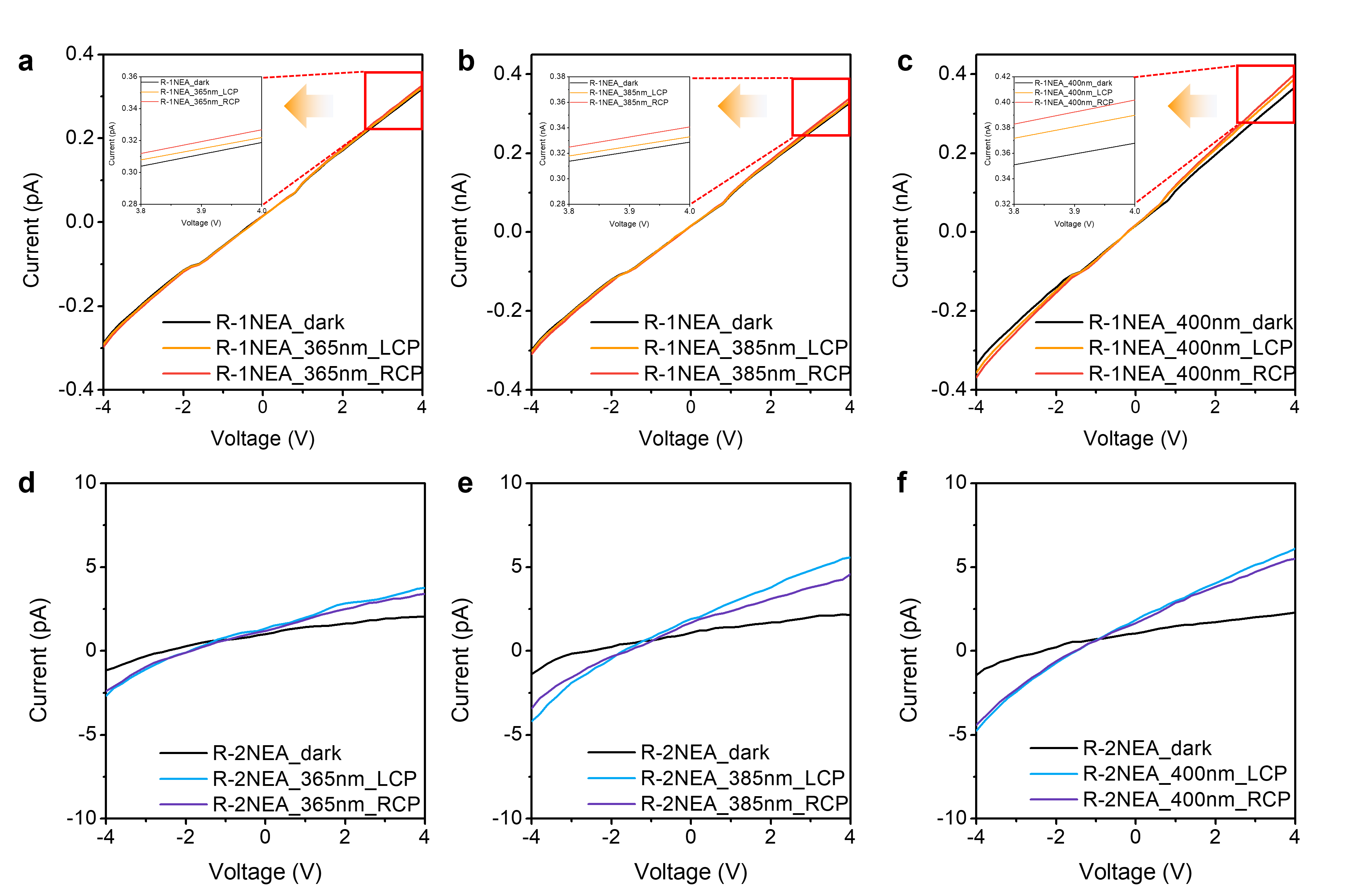


**Supplementary Figure 22.** **Photocurrent-voltage curve under LCP and RCP light with the varied CPL wavelength.** The small boxes show the zoomed-in photocurrent-voltage curve for clarity. Source data are provided as a Source Data file.

**
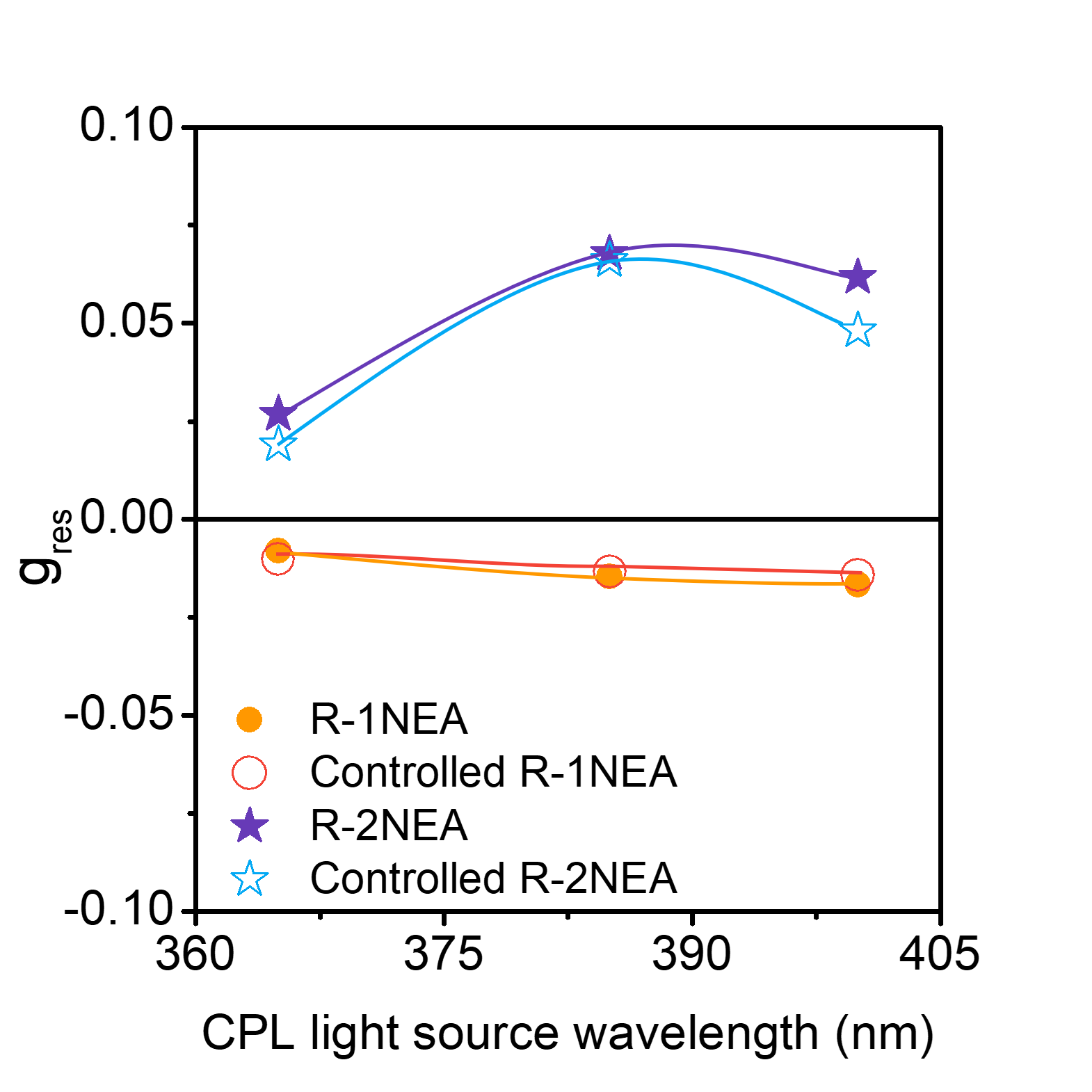
**

**Supplementary Figure 23. CPL light source wavelength dependent g_res_ of the CPL-PD.** The g_res_ were calculated using the flollowing eqation : g_res_ = $\frac{I_{L}-I_{R}}{{I_{L}+I}_{R}}$.


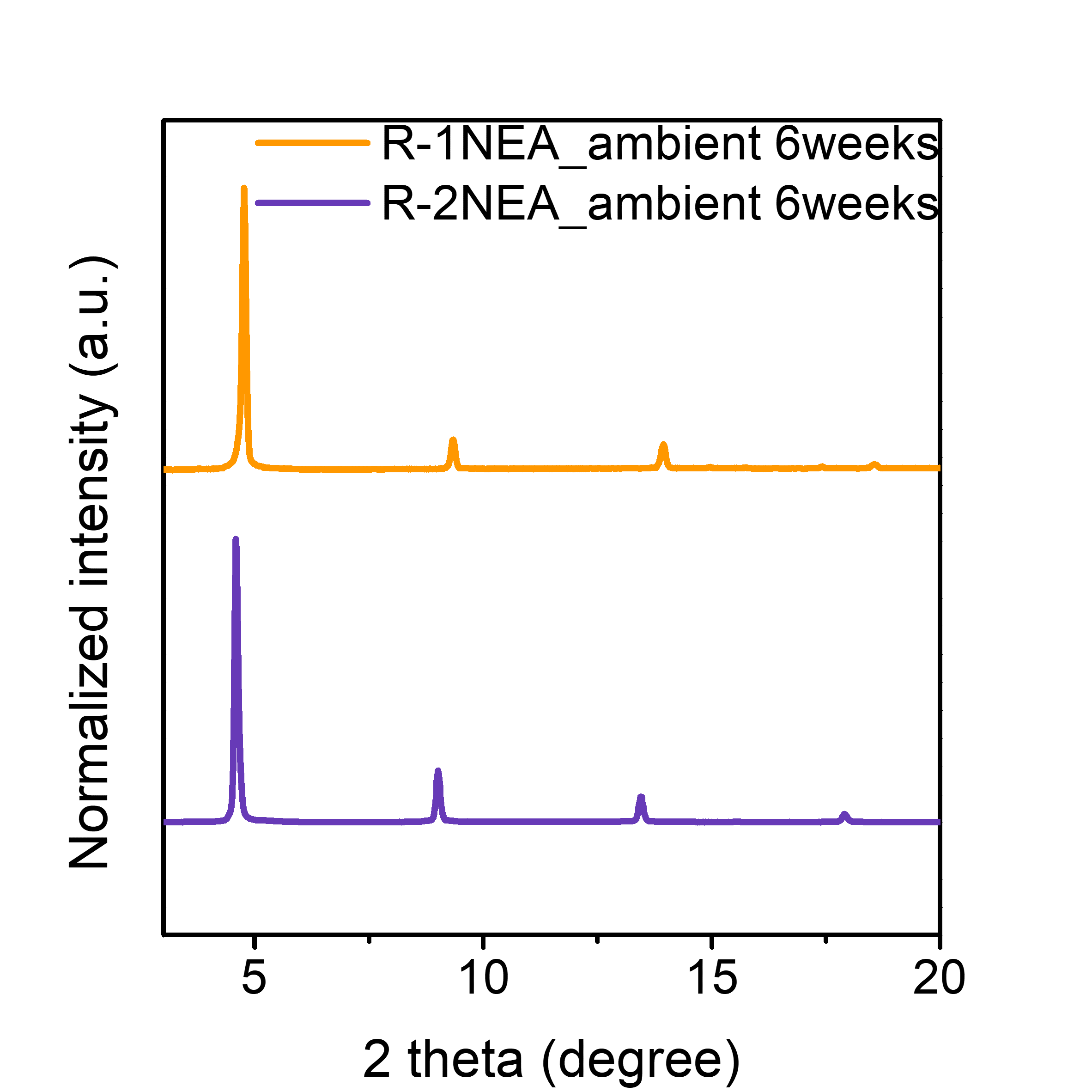


**Supplementary Figure 24.** XRD spectra of the NEA isomer OIHPs under ambient conditions; 20℃$\pm$3℃, RH 20%$\pm$5%. Source data are provided as a Source Data file.


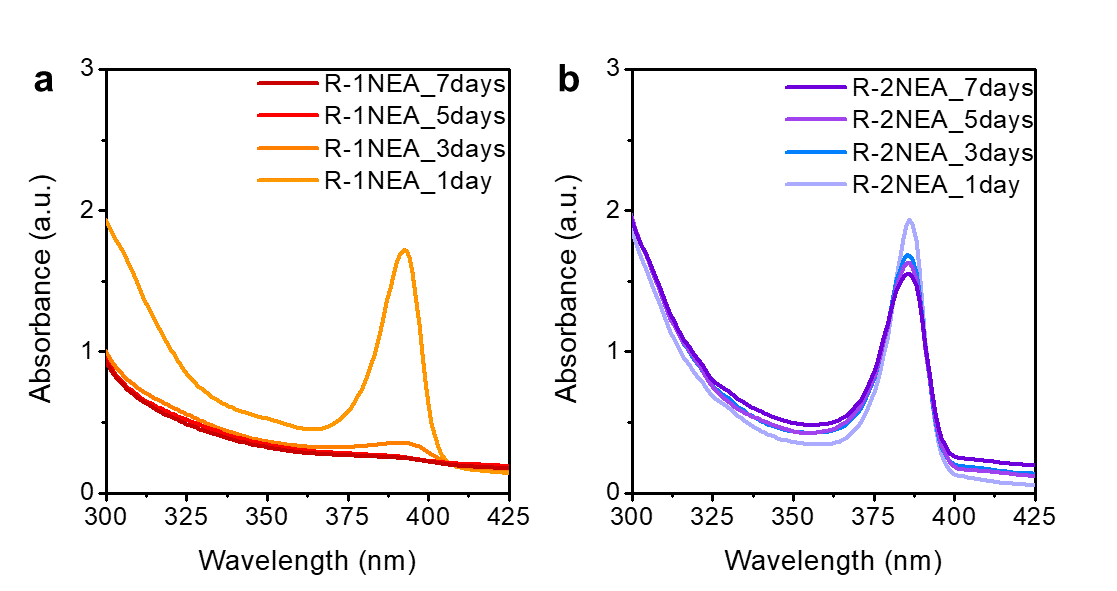


**Supplementary Figure 25. Absorbance spectra of the environmental stability test of the controlled NEA isomer OIHPs under harsh conditions for 7 days; 75℃ RH 75%.**  Tracing of absorbance spectra of a controlled R-1NEA thin-film and b controlled R-2NEA thin-film. Source data are provided as a Source Data file.


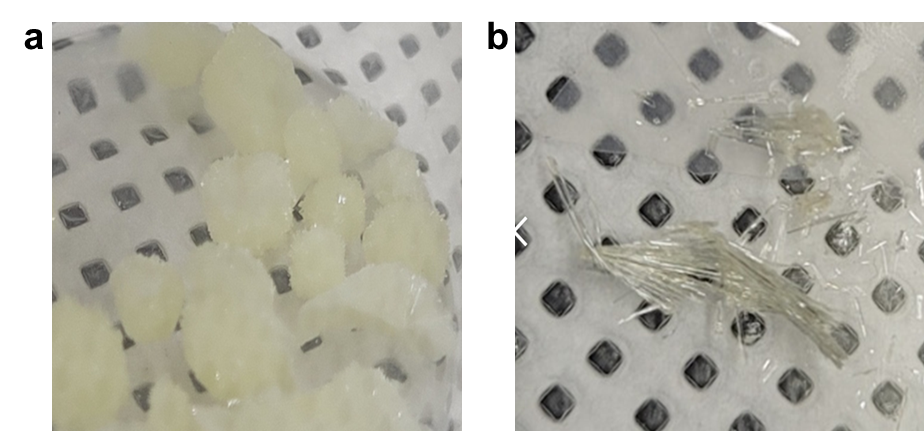


**Supplementary Figure 26. Photograph of synthesized (rac-2NEA)_2_PbBr_4_ crystals. a** Synthesized by AVC method, and b synthesized by ITC method. The crystals synthesized by AVC method exhibit opaque polycrystalline crystals that are unsuitable for SC-XRD.

**Supplementary Table 1**. Crystallographic data and structure refinement for (R-2NEA)_2_PbBr_4_.

| **Empirical formula** | C24 H28 Br4 N2 Pb |
| --- | --- |
| **Formula weight** | 871.31 |
| **Temperature** | 296(1) K |
| **Wavelength** | 0.71073 Å |
| **Crystal system** | Monoclinic |
| **Space group** | P21 |
| **Unit cell dimensions** | a = 8.7707(2) Å α= 90°  b = 7.8424(2) Å β= 99.469(2)°  c = 20.3546(5) Å γ = 90° |
| **Volume** | 1380.50(6) Å³ |
| **Z** | 2 |
| **Density (calculated)** | 2.095 Mg/m³ |
| **Absorption coefficient** | 11.909 mm⁻¹ |
| **F(000)** | 816 |
| **Crystal size** | 0.200 x 0.100 x 0.040 mm³ |
| **Theta range for data collection** | 2.354 to 25.999° |
| **Index ranges** | -10<=h<=10, -9<=k<=9, -25<=l<=25 |
| **Reflections collected** | 12224 |
| **Independent reflections** | 5329 [R(int) = 0.0460] |
| **Completeness to theta = 25.242** | 99.5 % |
| **Absorption correction** | Semi-empirical from equivalents |
| **Max. and min. transmission** | 0.65 and 0.37 |
| **Refinement method** | Full-matrix least-squares on F² |
| **Data / restraints / parameters** | 5329 / 9 / 285 |
| **Goodness-of-fit on F²** | 0.830 |
| **Final R indices [I>2sigma(I)]** | R1 = 0.0381, wR2 = 0.0899 |
| **R indices (all data)** | R1 = 0.0494, wR2 = 0.0980 |
| **Absolute structure parameter** | -0.016(13) |
| **Extinction coefficient** | n/a |
| **Largest diff. peak and hole** | 0.982 and -0.795 e.Å⁻³ |

**Supplementary Table 2**. Crystallographic data and structure refinement for (S-2NEA)_2_PbBr_4_.

| **Empirical formula** | C24 H28 Br4 N2 Pb |
| --- | --- |
| **Formula weight** | 871.31 |
| **Temperature** | 296(2) K |
| **Wavelength** | 0.71073 Å |
| **Crystal system** | Monoclinic |
| **Space group** | P21 |
| **Unit cell dimensions** | a = 8.7765(2) Å α= 90°  b = 7.8388(2) Å β= 99.4790(10)°  c = 20.3440(5) Å γ = 90° |
| **Volume** | 1380.50(6) Å³ |
| **Z** | 2 |
| **Density (calculated)** | 2.096 Mg/m³ |
| **Absorption coefficient** | 11.913 mm⁻¹ |
| **F(000)** | 816 |
| **Crystal size** | 0.160 x 0.160 x 0.080 mm³ |
| **Theta range for data collection** | 2.404 to 26.000° |
| **Index ranges** | -10<=h<=10, -9<=k<=9, -23<=l<=25 |
| **Reflections collected** | 19576 |
| **Independent reflections** | 5109 [R(int) = 0.0450] |
| **Completeness to theta = 25.242** | 97.5 % |
| **Absorption correction** | Semi-empirical from equivalents |
| **Max. and min. transmission** | 0.45 and 0.24 |
| **Refinement method** | Full-matrix least-squares on F² |
| **Data / restraints / parameters** | 5109 / 75 / 285 |
| **Goodness-of-fit on F²** | 0.969 |
| **Final R indices [I>2sigma(I)]** | R1 = 0.0348, wR2 = 0.0616 |
| **R indices (all data)** | R1 = 0.0467, wR2 = 0.0652 |
| **Absolute structure parameter** | -0.001(11) |
| **Extinction coefficient** | n/a |
| **Largest diff. peak and hole** | 1.480 and -0.731 e.Å⁻³ |

**Supplementary Table 3**. Crystallographic data and structure refinement for (Rac-2NEA)_2_PbBr_4_.

| **Empirical formula** | C24 H28 Br4 N2 Pb |
| --- | --- |
| **Formula weight** | 871.31 |
| **Temperature** | 296(2) K |
| **Wavelength** | 0.71073 Å |
| **Crystal system** | Monoclinic |
| **Space group** | P21/c |
| **Unit cell dimensions** | a = 20.1251(2) Å α= 90°  b = 7.91220(10) Å β= 92.5220(10)°  c = 8.74320(10) Å γ = 90° |
| **Volume** | 1390.86(3) Å³ |
| **Z** | 2 |
| **Density (calculated)** | 2.096 Mg/m³ |
| **Absorption coefficient** | 11.825 mm⁻¹ |
| **F(000)** | 816 |
| **Crystal size** | 0.200 x 0.060 x 0.020 mm³ |
| **Theta range for data collection** | 2.767 to 27.875° |
| **Index ranges** | -26<=h<=26, -10<=k<=10, -11<=l<=11 |
| **Reflections collected** | 23366 |
| **Independent reflections** | 3265 [R(int) = 0.0400] |
| **Completeness to theta = 25.242** | 98.6 % |
| **Absorption correction** | Semi-empirical from equivalents |
| **Max. and min. transmission** | 0.80 and 0.45 |
| **Refinement method** | Full-matrix least-squares on F² |
| **Data / restraints / parameters** | 3265 / 1 / 145 |
| **Goodness-of-fit on F²** | 1.035 |
| **Final R indices [I>2sigma(I)]** | R1 = 0.0276, wR2 = 0.052 |
| **R indices (all data)** | R1 = 0.0367, wR2 = 0.0555 |
| **Extinction coefficient** | n/a |
| **Largest diff. peak and hole** | 1.214 and -0.385 e.Å⁻³ |

**Supplementary Table 4. Fitting parameters of TRPL decay of NEA isomer OIHP thin films.**

|  | **A1 (%)** | **τ_1_** | **A_2_ (%)** | **τ_2_** | **Adj. R^2^** |
| --- | --- | --- | --- | --- | --- |
| **R-1NEA** | 97.08 | 0.280 | 2.92 | 2.068 | 0.992 |
| **Controlled R-1NEA** | 97.86 | 0.289 | 2.14 | 2.794 | 0.99 |
| **R-2NEA** | 65.60 | 0.519 | 34.40 | 1.985 | 0.996 |
| **Controlled R-2NEA** | 62.66 | 0.47 | 37.34 | 1.857 | 0.997 |

**Supplementary References**

1. Ahn, J. *et al*. A new class of chiral semiconductors: chiral-organic-molecule-incorporating organic–inorganic hybrid perovskites. *Mater. Horiz*. **4,** 851-856 (2017).

2. Ben,-M. *et al*. Probing the interaction of quantum dots with chiral capping molecules using circular dichroism spectroscopy. *Nano Lett.* 16, 7467-7473 (2016).

3. Ma, S. *et al.* Elucidating the origin of chiroptical activity in chiral 2D perovskites through nano-confined growth. *Nat. Commun.* 13, 3259 (2022).
